# Association between kidney stones and major adverse cardiovascular events: a population-based analysis

**DOI:** 10.1007/s00240-026-02028-8

**Published:** 2026-07-30

**Authors:** Ashton Smelser, Kang Liu, Steven Chi-Ho Leung, Chi-Fai NG

**Affiliations:** 1 Department of Neurology, University of Texas Southwestern Medical Center, Texas Dallas, USA; 2 Department of Urology, Urology Research Institute, The First Affiliated Hospital, Fujian Medical University, Fuzhou, 350005 China; 3 Department of Urology, Binhai Campus of the First Affiliated Hospital, National Regional Medical Center, Fujian Medical University, Fuzhou, 350212 China; 4 S.H. Ho Urology Centre, Department of Surgery, Faculty of Medicine, The Chinese University of Hong Kong, 4/F LCW Clinical Sciences Building, Prince of Wales Hospital, Shatin, Hong Kong, China

**Keywords:** Kidney stones, Myocardial infarction, Heart failure, Stroke, MACE

## Abstract

Kidney stones are an increasingly common condition, and evidence suggests a link between stone formation and cardiovascular risk. This study aimed to investigate the association between kidney stones and major adverse cardiovascular events (MACE), including acute myocardial infarction (AMI), heart failure (HF), and stroke. This multicenter retrospective study included 6,660 patients with newly diagnosed stone disease from 2013 to 2017 (KS group) and 19,980 patients without stone disease (NKS group) in the same period. The history of kidney stones was found to be significantly associated with the risk of a major adverse cardiovascular event (aORs: 1.20, 95% CIs: 1.07–1.36, *p* = 0.003). On multivariable Cox regression analysis, the kidney stone formers had an increased relative risk in stroke (aHRs: 1.21, 95%CIs: 1.02–1.42, *p* = 0.03) and AMI (aHRs: 1.57, 95%CIs: 1.28–1.92, *p* < 0.001), but not HF (aHRs: 1.11, 95%CIs: 0.92–1.34, *p* = 0.3). In contrast, in the female subgroup, kidney stones were associated with an increased risk of HF (aHRs: 1.46, 95% CIs: 1.10–1.93, *p* = 0.009). The kidney stone formers had an increased risk in stroke and acute myocardial infarction, while only female patients with kidney stones had an increased risk of heart failure.

**Supplementary Information:**

The online version contains supplementary material available at 10.1007/s00240-026-02028-8.

## Introduction

Cardiovascular disease accounts for approximately one-third of global deaths, with ischaemic heart disease and stroke being the most significant contributors that account for a combined 16 million deaths in 2020 [1, 2]. Heart failure (HF) represents an additional challenge for global cardiovascular health, affecting an estimated 64 million people [3, 4]. These major adverse cardiovascular events (MACE) are also among the primary drivers of global morbidity, with total worldwide costs projected to exceed US$1 trillion annually by 2030 [1, 3]. Moreover, China alone is estimated to have suffered a loss of US$558 billion in national income from 2005 to 2015 due to premature deaths linked to ischemic heart disease, stroke, and diabetes [5].

Countries across Asia have experienced rising rates of cardiovascular events and their sequelae in recent years, primarily due to the increase in cardiovascular risk factors among younger and middle-aged populations [6]. Hong Kong underwent urbanisation decades earlier than many other Asian countries, resulting in the early westernisation of the local lifestyle and heightened cardiovascular risk [7]. Given the prevalence, economic impacts, and associated morbidity and mortality of MACE on a global scale, identifying predictive conditions has become increasingly crucial.

Kidney stones are a common condition in the general population. A review of epidemiological data from seven countries has found incidence rates of 114–720 per 100,000 individuals and prevalence rates of 1.7–14.8%, which is seen to be rising in nearly all countries across sex, age, and race [8]. Several epidemiologic studies have demonstrated a link between kidney stone formation and systemic conditions, including ischemic heart disease, hypertension, chronic kidney disease, atherosclerosis, diabetes, obesity, and metabolic syndrome [9–11]. All of the conditions mentioned above are known risk factors for cardiovascular disease, and current evidence suggests that kidney stones, ischaemic heart disease, and stroke may share several underlying risk factors and pathophysiological mechanisms [12, 13].

In recent years, the increasing recognition of kidney stones as a systemic disorder has prompted greater efforts to examine their relationship with MACEs. Contemporary studies generally agree that a positive association exists between kidney stones and cardiovascular risk, although evidence on sex- and age-specific differences remains inconclusive. Acute myocardial infarction (AMI) and stroke have been the primary focus of existing studies, while the association with heart failure has received less attention. Moreover, most large-scale data come from Western cohorts, and Asian population-based evidence is comparatively limited. In this study, we aim to provide real-world evidence on the association between kidney stones and MACE via a large cohort.

## Method

This retrospective cohort study received approval from the institutional ethics committee. All data were sourced from the Clinical Data Analysis and Reporting System (CDARS), a pre-existing, population-based electronic database of patients using public health institutions across Hong Kong, China. MACE diagnoses were documented using the International Classification of Diseases, Ninth Revision (ICD-9) codes, irrespective of the year of entry. Previous studies have shown excellent coding accuracy within CDARS [14, 15].

### Study patients

Patients who had a first diagnosis of kidney stones (ICD-9 592) were identified from CDARS. The inclusion criteria were: (1) Seen in a public health institution in Hong Kong between January 1, 2013, and December 31, 2017, (2) Only patients with their first-time diagnosis of kidney stone during the study period were included, (3) aged 20 years or older at the time of their first-time diagnosis of kidney stone (index date). We chose an age cutoff of 20 years because kidney stones are extremely rare in individuals under 20 in our population, and inclusion of such cases would not meaningfully contribute to the analysis. The exclusion criteria were: (1) History of MACE before or within the 10 days following their index date, We applied a 10‑day washout period to exclude pre‑existing or peri‑diagnostic MACE events that were likely unrelated to incident kidney stones, thereby minimizing reverse causality; (2) Patients with a history of stone events prior to the study period. From the database, 6,660 patients with newly diagnosed stone disease (Supplementary Fig. 1) were identified.

A control cohort was randomly selected from patients without a history of kidney stones who were seen in outpatient clinics across Hong Kong during the first weeks of January and July from 2013 to 2017, with the first clinic visit documented within the study period acting as the index date. From the database, 1,734,140 patients were identified. Since the CDARS system can extract data from a maximum of only 60,000 patients at a time, we randomly selected 60,000 patients from a total of 1,734,140 as the control group.

All patients were followed until the date of death or 31 January 2023, whichever occurred first.

### Data collection

First diagnosis of AMI, stroke, and HF were the primary outcomes of interest and were identified using ICD-9 codes exclusively. Comorbidities measured at baseline included the presence of chronic kidney disease, pulmonary disease, connective tissue disease, dementia, diabetes, hemiplegia, HIV, hypertension, hyperlipidemia, liver disease, cancer, peptic ulcer disease, and peripheral vascular disease. A complete list of ICD-9 codes within each category of comorbidities can be found in the supplemental material (Supplementary Table 1). No laboratory variables were used in the study as these are not readily available in CDARS. Similarly, no information on body mass index, smoking status, alcohol consumption, physical activity, or dietary habits could be retrieved, because these variables are not stored in a structured format within the hospital information system. Demographic information, including age and sex, was also recorded.

### Statistical analyses

All statistical analyses were performed using R software (version 4.2.0; R Foundation for Statistical Computing, Vienna, Austria).

To minimize confounding by indication, we constructed a matched cohort using propensity score matching. Patients with a history of kidney stones (KS group) were matched in a 1:3 ratio to those without kidney stones (NKS group) via the “MatchIt” package, without replacement and based on a nearest-neighbor algorithm. Matching covariates included sex, age, index year, and the following comorbidities: chronic kidney disease, pulmonary disease, connective tissue disease, dementia, diabetes, hemiplegia, HIV infection, hypertension, hyperlipidemia, liver disease, cancer, peptic ulcer disease, and peripheral vascular disease. Given the observational nature of this study, we acknowledge that unmeasured confounding remains a potential concern even after propensity score matching and multivariable adjustment. Specifically, our database did not capture lifestyle and anthropometric factors such as body mass index [16], smoking status [17], alcohol consumption [18], physical activity, or dietary habits [19], all of which are known to influence both nephrolithiasis and cardiovascular outcomes. Because these unmeasured factors are likely to be more prevalent or unfavorable in kidney stone formers, their absence would tend to produce residual positive confounding, potentially biasing our effect estimates away from the null (i.e., overestimating the true independent association). Therefore, while our analytical strategy minimized measured confounding, the results should be interpreted as associational, with a pre-specified acknowledgment that the reported hazard ratios may reflect a composite effect of stone disease and its associated lifestyle/metabolic milieu rather than a pure causal effect.

Baseline characteristics between the matched KS and NKS cohorts were compared. Categorical variables are presented as frequencies (percentages) and were analyzed using Pearson’s chi-square test. Continuous variables are summarized as medians with interquartile ranges (IQRs) and were compared using the Mann-Whitney U test due to non-normal distributions.

We employed multivariable logistic regression to estimate cumulative MACE odds and Cox regression to leverage time-to-event data for individual components, with the two approaches serving complementary roles. The independent association between kidney stones and the composite outcome of MACE was assessed using multivariable logistic regression. Results are reported as adjusted odds ratios (aORs) with corresponding 95% confidence intervals (CIs). The model adjusted for the full set of covariates used in the propensity score matching to address residual confounding.

For time-to-event endpoints, we performed Kaplan-Meier survival analysis, and between-group differences were assessed using the log-rank test. To quantify the risk for individual MACE components (AMI, stroke, HF), multivariable Cox proportional hazards regression models were fitted to calculate adjusted hazard ratios (aHRs) and 95% CIs. These models adjusted for the same covariates as the primary logistic model. Prespecified subgroup analyses were conducted to explore potential effect modification by sex and age groups.

## Results

### Baseline characteristics

During the study period from 2013 to 2017, we identified 6,660 patients with newly diagnosed kidney stones as the case group (KS group) (Supplementary Fig. 1). Using propensity score matching (at a 1:3 ratio), each case was matched to three patients without kidney stones from the same period, resulting in a matched cohort of 26,640 individuals (6,660 cases and 19,980 matched controls). The median follow-up duration was 7.4 years for the KS group and 8.1 years for the NKS group. Table 1 summarized the baseline characteristics of the two groups. After propensity score matching, the standardized mean differences for all observed covariates were reduced to below 0.1, indicating substantial improvement in balance between the two groups. There were no significant differences between KS and NKS groups in terms of age, gender, index year, and all baseline comorbidities (all standardized mean differences less than 0.1). During the follow‑up period, MACE occurred in 431 patients (6.5%) in the KS group and 1,120 patients (5.6%) in the NKS group.

**Table 1:** Baseline characteristics between No Kidney Stone and Kidney Stone groups after propensity score matching

Characteristics	No Kidney Stone *n* = 19,980	Kidney Stone *n* = 6660	Standard Mean Difference
**Age**,** median [IQR]**	56 [46–65]	56 [47–65]	0.015
**Male Gender**,** n (%)**	11,526 (58)	3768 (57)	0.022
**Index Year**			
2013	4570 (23)	1445 (22)	0.029
2014	3832 (19)	1282 (19)	0.0018
2015	3934 (20)	1323 (20)	0.0044
2016	3822 (19)	1308 (20)	0.013
2017	3822 (19)	1302 (19)	0.011
**Chronic Kidney Disease**	120 (0.6)	48 (0.7)	0.014
**Pulmonary Disease**	486 (2.4)	218 (3.3)	0.047
**Connective Tissue Disease**	109 (0.5)	51 (0.8)	0.025
**Dementia**	351 (1.8)	145 (2.2)	0.029
**Diabetes**	1605 (8.0)	613 (9.2)	0.041
**Hemiplegia**	8 (0.1)	6 (0.1)	0.017
**HIV/AIDS**	17 (0.1)	7 (0.1)	0.0062
**Hypertension**	2983 (15)	1071 (16)	0.031
**Hyperlipidemia**	635 (3.2)	271 (4.1)	0.045
**Liver Disease**	342 (1.7)	143 (2.1)	0.030
**Cancer**	987 (4.9)	363 (5.5)	0.023
**Peptic Ulcer Disease**	1017 (5.1)	410 (6.2)	0.044
**Peripheral Vascular Disease**	96 (0.5)	41 (0.6)	0.017

### Outcomes

The multivariable logistic regression analysis revealed a significant association between kidney stones and MACE. The adjusted odds ratio was 1.20 (95% CIs: 1.07–1.36, *p* = 0.003) (Table 2).

**Table 2:** Multivariable logistic regression analysis

Group	*n*	MACE	aORs (95% CI)	*p* value
Kidney Stone				
No	19,980	1120	Ref	
Yes	6660	431	**1.20 (1.07–1.36)**	**0.003**

**Fig. 1:**
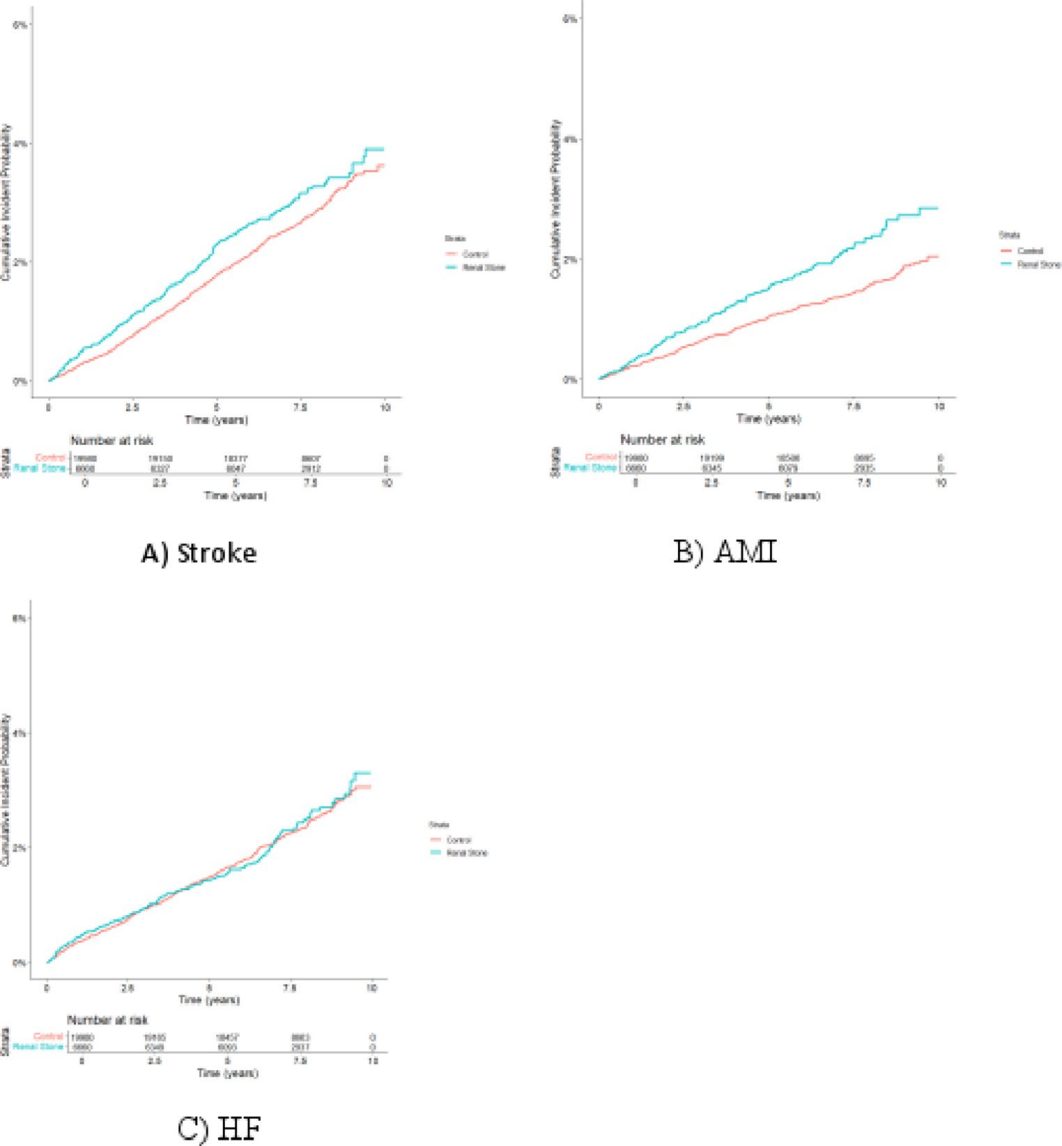
Survival outcomes. **A**) Stroke, **B**) AMI, **C**) HF

Based on Kaplan-Meier curves with log-rank testing, the cumulative incidence of AMI was significantly higher in the KS group compared to the NKS group (*p* < 0.001). In contrast, the cumulative incidence curves for stroke (*p* = 0.11) and HF (*p* = 0.9) showed no statistically significant divergence between the two groups (Fig. 1).

Multivariable Cox regression analysis demonstrated that kidney stone was an independent risk factor for stroke (aHRs: 1.21, 95%CIs: 1.02–1.42, *p* = 0.03) and AMI (aHRs: 1.57, 95%CIs: 1.28–1.92, *p* < 0.001), but not HF (aHRs: 1.11, 95%CIs: 0.92–1.34, *p* = 0.3) (Table 3).

**Table 3:** Multivariable Cox regression analysis of Stroke, AMI and HF

Group	*n*	Events	aHRs (95% CIs)	*p* value
**Stroke**				
No Kidney Stone	19,980	522	Ref	
Kidney Stone	6660	194	**1.21 (1.02–1.42)**	**0.03**
**AMI**				
No Kidney Stone	19,980	289	Ref	
Kidney Stone	6660	139	**1.57 (1.28–1.92)**	**< 0.001**
**HF**				
No Kidney Stone	19,980	440	Ref	
Kidney Stone	6660	144	1.11 (0.92–1.34)	0.3

### Subgroup analysis

Compared to male patients without kidney stones, male kidney stone patients had a significantly elevated risk of both stroke (aHRs: 1.34, 95% CIs: 1.09–1.64, *p* = 0.005) and AMI (aHRs: 1.65, 95% CIs: 1.29–2.11, *p* < 0.001). The risk of HF, however, was comparable between the groups (aHRs: 0.89, 95% CIs: 0.68–1.15, *p* = 0.37). In contrast, in the female subgroup, kidney stones were associated with an increased risk of HF (aHRs: 1.46, 95% CIs: 1.10–1.93, *p* = 0.009), but not with stroke (aHRs: 0.99, 95% CIs: 0.75–1.32, *p* = 0.9) or AMI (aHRs: 1.30, 95% CIs: 0.89–1.90, *p* = 0.18) (Table 4).

**Table 4:** Multivariable Cox regression analysis of sex subgroups

MACE type	Sex	Group	*n*	Events	aHRs (95% CIs)	*p* value
**Stroke**	**Female**	No Kidney Stone	8454	199	Ref	
		Kidney Stone	2892	65	0.99 (0.75–1.32)	0.9
	**Male**	No Kidney Stone	11,526	323	Ref	
		Kidney Stone	3768	129	**1.34 (1.09–1.64)**	**0.005**
**AMI**	**Female**	No Kidney Stone	8454	89	Ref	
		Kidney Stone	2892	40	1.30 (0.89–1.90)	0.18
	**Male**	No Kidney Stone	11,526	200	Ref	
		Kidney Stone	3768	99	**1.65 (1.29–2.11)**	**< 0.001**
**HF**	**Female**	No Kidney Stone	8454	166	Ref	
		Kidney Stone	2892	74	**1.46 (1.10–1.93)**	**0.009**
	**Male**	No Kidney Stone	11,526	274	Ref	
		Kidney Stone	3768	70	0.89 (0.68–1.15)	0.37

In the age-stratified analysis, the association between kidney stones and cardiovascular risk varied across age groups. Patients with kidney stones in the 20–45 age group had a significantly higher risk of stroke (aHRs: 2.41, 95% CIs: 1.27–4.57, *p* = 0.007) compared to their non-kidney stone counterparts, but their risks of AMI (aHRs: 1.49, 95% CIs: 0.60–3.69, *p* = 0.4) and HF (aHRs: 1.08, 95% CIs: 0.28–4.10, *p* = 0.9) were comparable. In the 46–70 age group, kidney stones were associated with an increased risk of AMI (aHRs: 1.89, 95% CIs: 1.44–2.46, *p* < 0.001), but not with stroke or HF. Among patients older than 70 years, stone formers did not have a significantly higher risk of stroke, AMI, or HF compared to non-stone formers (Table 5).

**Table 5:** Multivariable Cox regression analysis of age subgroups

MACE type	Age	Group	*n*	Events	aHRs (95% CIs)	*p* value
**Stroke**	**20–45**	No Kidney Stone	4694	21	Ref	
		Kidney Stone	1502	**18**	**2.41 (1.27–4.57)**	**0.007**
	**46–70**	No Kidney Stone	12,362	273	Ref	
		Kidney Stone	4218	103	1.12 (0.89–1.40)	0.3
	**> 70**	No Kidney Stone	2924	228	Ref	
		Kidney Stone	940	73	1.11 (0.85–1.45)	0.5
**AMI**	**20–45**	No Kidney Stone	4694	15	Ref	
		Kidney Stone	1502	7	1.49 (0.60–3.69)	0.4
	**46–70**	No Kidney Stone	12,362	142	Ref	
		Kidney Stone	4218	**88**	**1.89 (1.44–2.46)**	**< 0.001**
	**> 70**	No Kidney Stone	2924	132	Ref	
		Kidney Stone	940	44	1.15 (0.82–1.63)	0.4
**HF**	**20–45**	No Kidney Stone	4694	9	Ref	
		Kidney Stone	1502	3	1.08 (0.28–4.10)	0.9
	**46–70**	No Kidney Stone	12,362	145	Ref	
		Kidney Stone	4218	68	1.35 (1.01–1.81)	0.044
	**> 70**	No Kidney Stone	2924	286	Ref	
		Kidney Stone	940	73	0.94 (0.72–1.21)	0.6

## Discussion

Our study revealed that individuals with a history of kidney stones were at an elevated risk for MACE. When individual MACE components were examined, kidney stones were significantly associated with an increased risk of stroke and AMI, but not HF, after adjustment for multiple confounders. The association between kidney stone history and subsequent MACE remained robust in multivariable Cox regression models. Although the risk estimates varied according to event type, age, and sex, these findings extend the existing literature by providing population-based Asian data on the association between nephrolithiasis and cardiovascular outcomes.

Several biologically plausible mechanisms may underlie the observed association between kidney stones and MACE. First, nephrolithiasis is increasingly recognized as a systemic inflammatory condition; elevated circulating pro-inflammatory cytokines (e.g., IL-6, TNF-α) and acute-phase reactants (e.g., CRP) have been documented in stone formers, and systemic inflammation is a well-established driver of atherosclerosis and plaque instability. Second, oxidative stress, which promotes both crystal deposition in renal tubules and endothelial injury, may represent a shared pathogenic pathway linking the two diseases [20]. Third, urinary stone formation often coexists with metabolic derangements, including insulin resistance, dyslipidemia, and hypertension, that collectively contribute to vascular damage. Fourth, emerging evidence suggests that gut microbiome alterations and chronic low-grade endotoxemia may play a mediating role [21]. While these mechanisms are speculative in the context of our observational data, they provide a framework for interpreting the epidemiological association and warrant further investigation.

We found that individuals with a history of kidney stones had an AMI risk 1.57-fold higher than their NKS counterparts. Reports of a suspected association between stone disease and cardiovascular disease extend as far back as the 1970s, with Elmfeldt et al. being among the first to examine the relationship between self-reported history of kidney stones amongst 270 survivors of myocardial infarction and a sample of the general population in Göteborg, Sweden in 1976 [22]. The latest evidence supports a well-established association between kidney stone disease and MACE [10, 11]. A meta-analysis of 6 articles, including over 49.5 thousand stone patients and 3.5 million controls with a median follow-up of 8.9 years, also confirmed this finding that kidney stones were associated with a 1.29-fold higher risk of MI, as well as the need for coronary revascularisation [12].

Stroke risk among individuals with a history of kidney stones was also significantly increased in our study, which aligns with existing literature from multiple countries. Domingos and Serra analysed 23,349 responses from the Portuguese National Health Survey, finding that those who had previously formed stones were 1.33 times more likely to have experienced a stroke than those who had not. However, this association did not reach significance after adjusting for comorbidities [23]. It is also important to note that this study relied on self-reported events, which could have affected their results. Another study from Taiwan indicated that individuals with a history of stone formation were at an increased risk of stroke, particularly ischaemic stroke, as documented in a meta-analysis of eight additional articles [24]. Despite our study having a sufficiently large sample size, the specific type of stroke (ischaemic vs. haemorrhagic) was not adequately documented for analysis, preventing us from confirming this association.

Few studies have specifically examined the association between kidney stone disease and heart failure. In our cohort, female patients with a history of kidney stones showed an increased risk of HF. This finding is consistent with the observation that several risk factors for HF — including hypertension, hyperlipidemia, obesity, smoking, and diabetes — are also well-established risk factors for kidney stone formation [25]. Lin et al. reported that kidney stone presence was associated with an increased risk of ICU treatment and HF following acute AMI [26]. Whether this reflects a direct role of stone disease in HF development, is mediated by AMI, or merely reflects shared cardiometabolic risk factors cannot be determined from our data, as we did not examine the temporal sequence of AMI and HF diagnoses in this cohort. We also note that patients with stone disease are routinely advised to increase fluid intake to prevent recurrence; however, the safety of this recommendation in individuals with volume-sensitive conditions such as HF has not been well studied [7], and future research is needed to explore this potential clinical tension.

Existing literature suggests a differential association by sex between stone formation and the risks of AMI and stroke. Analysis of women from the Nurse’s Health Studies I and II, and men from the Health Professionals Follow-up Study with a history of stone disease, demonstrated a higher risk of myocardial infarction and coronary revascularisation across all age groups in two distinct female cohorts, but found no such association in male cohorts [27]. A 2019 study involving 22,636 stone formers and 90,544 controls from Korea further clarified these associations, finding that young women and middle-aged men in the stone group were at the highest risk [28]. Additionally, Asian populations, and Chinese men specifically, experience higher incidence and mortality rates of stroke than their female counterparts [29, 30].

The incidence of both kidney stones and MACE can vary with age. Our results indicate that stone formers in the young age group (aged 20–40) have an increased risk of stroke, while those aged 46–70 face an elevated risk of acute AMI. This largely aligns with previous findings regarding the link between kidney stones and stroke, which have demonstrated that the risk of stroke is highest among younger stone formers. Additionally, the AMI risk in stone formers shows a varied association by age, with earlier studies identifying an increased risk in individuals under 50, which diminishes thereafter and disappears entirely after age 70 [11]. Our observation of an 89% increased AMI risk in those aged 46–70, with no significant rise in risk for individuals over 70, corroborates these findings.

### Strengths and limitations

The strengths of the study include a large cohort of patients from hospitals across Hong Kong, gathered over a period of ten years. Furthermore, CDARS serves as a centralised source of information, ensuring consistent reporting and high coding accuracy.

However, several important limitations should be acknowledged. First, as a retrospective study encompassing multiple institutions, there remains the possibility of misdiagnosis, miscoding, missing data, or variations in physician preferences. Second, information on key lifestyle and socioeconomic factors, including body mass index, smoking, alcohol consumption, physical activity, dietary habits, and socioeconomic status, was not available in our database. These factors are known to affect both kidney stone formation and cardiovascular risk, and their absence may have led to residual confounding and incomplete matching between cohorts. Notably, because these unmeasured lifestyle and anthropometric factors tend to be more unfavorable in stone formers (e.g., higher rates of obesity [16], sedentary behavior, and high-sodium diet [19]), their omission likely produces residual positive confounding. This means our reported hazard ratios may overestimate the true independent contribution of kidney stone disease per se. If these factors had been available for adjustment, the observed associations—for instance, the aHR of 1.57 for AMI—would probably be attenuated toward a more modest estimate, although the direction of the association is unlikely to be reversed. Third, the lack of laboratory biomarkers (e.g., renal function, lipid profiles, glycemic control) and detailed medication records precluded us from controlling for disease severity and cardiometabolic management, which further limits causal interpretation. Fourth, our study is limited by the lack of stone composition data. Nephrolithiasis is a metabolically heterogeneous condition; uric acid stones exhibit strong correlations with hyperuricemia, metabolic syndrome, and insulin resistance [31], while calcium oxalate stones do not share these associations to the same extent. By aggregating all stone types, our estimates may predominantly reflect the cardiovascular risk profile of the high-risk uric acid stone subgroup. This aggregation precludes us from determining whether the observed MACE association applies uniformly across all stone types, and warrants further research with stone composition analysis.

## Conclusion

In conclusion, this study contributes to the growing evidence that kidney stone disease is associated with subsequent cardiovascular risk. After adjustment for the covariates available in our dataset, we observed a significantly elevated risk for AMI and stroke among stone formers, while the association with heart failure was not significant in the overall cohort but emerged specifically in the female subgroup. These associations varied by sex and age group. Importantly, because key confounders — including BMI, smoking, physical activity, dietary habits, and socioeconomic status — were not available for adjustment, our findings should be interpreted as an association rather than a causal effect, and residual confounding cannot be excluded. Further studies incorporating detailed lifestyle and metabolic data are warranted to clarify these relationships and explore the clinical implications for risk stratification.

Data sharing statement.

Individual, de-identified participant data used in these analyses will be shared on request from any qualified investigator after the approval of a protocol and signed data access agreement via The Chinese University of Hong Kong (Hong Kong, China).

## Supplementary Information

Below is the link to the electronic supplementary material.

Supplementary Material 1

## Data Availability

Individual, de-identified participant data used in these analyses will be shared on request from any qualified investigator after the approval of a protocol and signed data access agreement via The Chinese University of Hong Kong (Hong Kong, China).

## References

[1] Khan MA, Hashim MJ, Mustafa H et al (2020) Global Epidemiology of Ischemic Heart Disease: Results from the Global Burden of Disease Study. Cureus 12:e9349 32742886 10.7759/cureus.9349 PMC7384703

[2] Tsao CW, Aday AW, Almarzooq ZI et al (2023) Heart Disease and Stroke Statistics-2023 Update: A Report From the American Heart Association. Circulation 147:e93–e621 36695182 10.1161/CIR.0000000000001123 PMC12135016

[3] Savarese G, Becher PM, Lund LH et al (2023) Global burden of heart failure: a comprehensive and updated review of epidemiology. Cardiovasc Res 118:3272–3287 35150240 10.1093/cvr/cvac013

[4] Shahim B, Kapelios CJ, Savarese G et al (2023) Global Public Health Burden of Heart Failure: An Updated Review. Card Fail Rev 9:e11 37547123 10.15420/cfr.2023.05 PMC10398425

[5] Katan M, Luft A (2018) Global Burden of Stroke. Semin Neurol 38:208–211 29791947 10.1055/s-0038-1649503

[6] Peng S, Liu X, Cao W et al (2023) Global, regional, and national time trends in mortality for stroke, 1990–2019: An age-period-cohort analysis for the global burden of disease 2019 study and implications for stroke prevention. Int J Cardiol 383:117–131 37150213 10.1016/j.ijcard.2023.05.001

[7] Cheungpasitporn W, Rossetti S, Friend K et al (2016) Treatment effect, adherence, and safety of high fluid intake for the prevention of incident and recurrent kidney stones: a systematic review and meta-analysis. J Nephrol 29:211–219 26022722 10.1007/s40620-015-0210-4 PMC4831051

[8] Romero V, Akpinar H, Assimos DG (2010) Kidney stones: a global picture of prevalence, incidence, and associated risk factors. Rev Urol 12:e86–96 20811557 PMC2931286

[9] Lange JN, Mufarrij PW, Wood KD et al (2012) The association of cardiovascular disease and metabolic syndrome with nephrolithiasis. Curr Opin Urol 22:154–159 22262248 10.1097/MOU.0b013e32834fc31f PMC8475956

[10] Lin SY, Lin CL, Chang YJ et al (2016) Association Between Kidney Stones and Risk of Stroke: A Nationwide Population-Based Cohort Study. Med (Baltim) 95:e2847 10.1097/MD.0000000000002847 PMC4779012 26937915

[11] Alexander RT, Hemmelgarn BR, Wiebe N et al (2014) Kidney stones and cardiovascular events: a cohort study. Clin J Am Soc Nephrol 9:506–512 24311706 10.2215/CJN.04960513 PMC3944758

[12] Liu Y, Li S, Zeng Z et al (2014) Kidney stones and cardiovascular risk: a meta-analysis of cohort studies. Am J Kidney Dis 64:402–410 24797522 10.1053/j.ajkd.2014.03.017

[13] Arafa A, Eshak ES, Iso H (2020) Oxalates, urinary stones and risk of cardiovascular diseases. Med Hypotheses 137:109570 31972450 10.1016/j.mehy.2020.109570

[14] Liu K, Zhao H, Chen X et al (2025) Association Between Hypoglycemia Agents and Long-term Survival Outcomes for Patients with Non-muscle-invasive Bladder Cancer Treated with Intravesical Bacillus Calmette-Guérin Immunotherapy. Eur Urol Oncol 8:164–170 39689991 10.1016/j.euo.2024.12.002

[15] Chan JSK, Tang P, Hui JMH et al (2022) Association between duration of gonadotrophin-releasing hormone agonist use and cardiovascular risks: A population-based competing-risk analysis. Prostate 82:1477–1480 35915869 10.1002/pros.24423 PMC9804360

[16] Carbone A, Al Salhi Y, Tasca A et al (2018) Obesity and kidney stone disease: a systematic review. Minerva Urol Nefrol 70:393–400 29856171 10.23736/S0393-2249.18.03113-2

[17] Li C, Engström G, Hedblad B et al (2005) Risk factors for stroke in subjects with normal blood pressure: a prospective cohort study. Stroke 36:234–238 15618439 10.1161/01.STR.0000152328.66493.0a

[18] de Lorimier AA (2000) Alcohol, wine, and health. Am J Surg 180:357–361 11137687 10.1016/s0002-9610(00)00486-4

[19] Manolis AA, Manolis TA, Vouliotis A et al (2025) Nephrolithiasis and Cardiovascular Disease. Cardiol Rev 10.1097/CRD.0000000000001149 41398712

[20] Muschialli L, Mannath A, Moochhala SH et al (2024) Epidemiological and biological associations between cardiovascular disease and kidney stone formation: A systematic review and meta-analysis. Nutr Metab Cardiovasc Dis 34:559–568 38431384 10.1016/j.numecd.2023.09.011

[21] Comellato G, Caletti C, Giani A et al (2024) Arterial stiffness and cardiovascular risk in patients with nephrolithiasis: a 10-year prospective study. J Hypertens 42:1358–1363 38934190 10.1097/HJH.0000000000003736

[22] Elmfeldt D, Wilhelmsen L, Wedel H et al (1976) Primary risk factors in patients with myocardial infarction. Am Heart J 91:412–419 1258747 10.1016/s0002-8703(76)80320-1

[23] Domingos F, Serra A (2011) Nephrolithiasis is associated with an increased prevalence of cardiovascular disease. Nephrol Dial Transpl 26:864–868 10.1093/ndt/gfq501 20709737

[24] Yuan M, Zhou HY, Hu F et al (2021) Association between kidney stones and risk of developing stroke: a meta-analysis. Neurol Sci 42:4521–4529 33606128 10.1007/s10072-021-05113-5 PMC8519881

[25] van Essen BJ, Voors AA, Tromp J (2022) Risk factors for the development of heart failure in patients with or without prior myocardial infarction. Eur J Heart Fail 24:985–987 35560757 10.1002/ejhf.2538

[26] Lin SK, Liu JM, Chang YH et al (2017) Increased risk of endotracheal intubation and heart failure following acute myocardial infarction in patients with urolithiasis: a nationwide population-based study. Ther Clin Risk Manag 13:245–253 28260911 10.2147/TCRM.S123702 PMC5328140

[27] Ferraro PM, Taylor EN, Eisner BH et al (2013) History of kidney stones and the risk of coronary heart disease. JAMA 310:408–415 23917291 10.1001/jama.2013.8780 PMC4019927

[28] Kim SY, Song CM, Bang W et al (2019) Nephrolithiasis predicts ischemic stroke: A longitudinal follow-up study using a national sample cohort. Int J Med Sci 16:1050–1056 31523166 10.7150/ijms.34417 PMC6743278

[29] Woo J, Ho SC, Goggins W et al (2014) Stroke incidence and mortality trends in Hong Kong: implications for public health education efforts and health resource utilisation. Hong Kong Med J 20:24–29 25001032

[30] Peng JP, Zheng H (2017) Kidney stones may increase the risk of coronary heart disease and stroke: A PRISMA-Compliant meta-analysis. Med (Baltim) 96:e7898 10.1097/MD.0000000000007898 PMC5572031 28834909

[31] Colin IM, Pozdzik A (2026) Obesity, Metabolic Syndrome, Diabetes and Kidney Stones: Strengthening Links Over Time? touchREV Endocrinol 22:29–36 42272999 10.17925/EE.2026.22.1.7 PMC13249147

