# Supplementary material for: Association between kidney stones and major adverse cardiovascular events: a population-based analysis
Source: Urolithiasis. 2026 Jul 30;54(1):128. doi: 10.1007/s00240-026-02028-8 (PMC13424457; doi:10.1007/s00240-026-02028-8)
Supplement: Supplementary file 1 — Supplementary Material 1 [file 240_2026_2028_MOESM1_ESM.docx]

Supplementary Figure 1. The selection of patients with newly diagnosed stone disease.


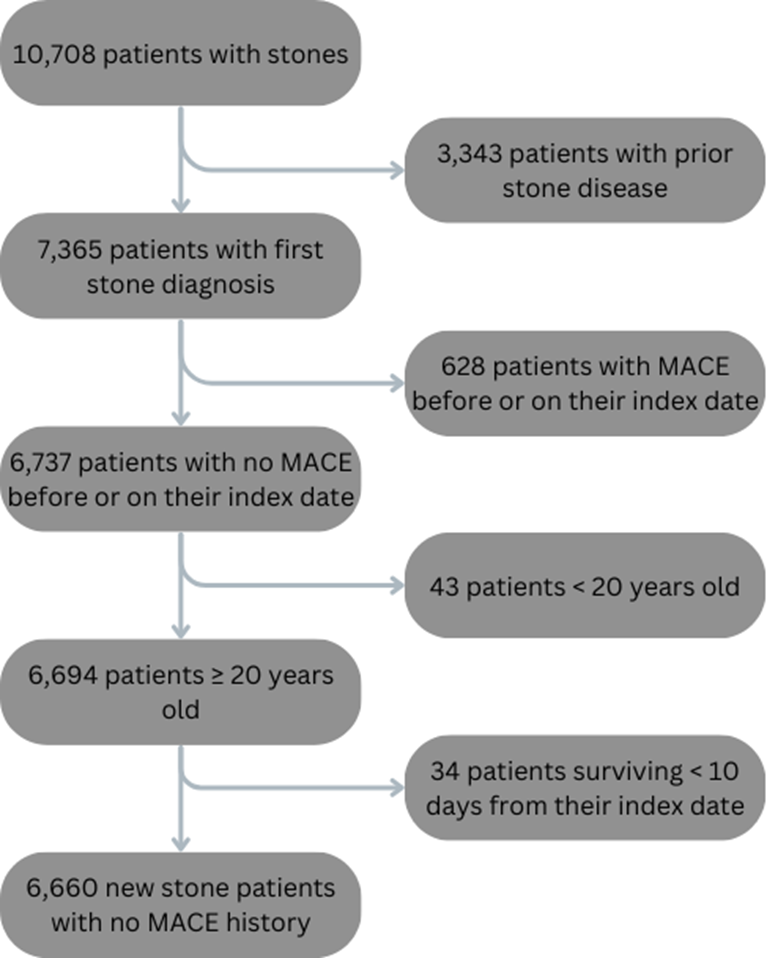


Supplementary Table 1. ICD-9-CM diagnosis codes of comorbidities.

| Diagnosis | ICD-9 Codes |
| --- | --- |
| Myocardial Infarction | 410 |
| Congestive Heart Failure | 398.91, 402.01, 402.11, 402.91, 404.01, 404.03, 404.11, 404.13, 404.91, 404.93, 428 |
| Stroke/Transient Ischemic Attack | 430, 431, 432, 433.01, 433.31, 433.81, 433.91, 434 - 437 |
| Chronic Kidney Disease | 440.1 (2), 585.9 (1 – 4, 9), 585 (1 – 2), 996.81, V42.0, V56, V58.49 (1), E878.0 (4) |
| Chronic Obstructive Pulmonary Disease | 490 - 496 |
| Autoimmune Disease | 446.0 (2), 446.4, 710, 714, 042, V08 |
| Cardiovascular Disease | 401 – 405, 437.2, 250.7, 441, 443, 785.4, V43.4 |
| Gastrointestinal Disease | 456, 571, 572, 531 – 534, 578 |
| Metabolic Disease | 272, 250 |
| Neurologic Disease | 290, 294, 331.1, 331.8, 332, 333.4, 340, 342 |
| Cancer | 140 - 208 |
